# Improving shared decision-making in bronchiectasis

**DOI:** 10.1136/bmjresp-2024-003049

**Published:** 2025-09-03

**Authors:** Paul McCallion, Judy M Bradley, Adam Lewis, Lisa Robinson, Joanne Lally, Anthony De Soyza

**Affiliations:** 1 Newcastle Upon Tyne Hospitals NHS Foundation Trust, Newcastle Upon Tyne, UK; 2 Newcastle University Population Health Sciences Institute, Newcastle Upon Tyne, UK; 3 Clinical Research Facility, Queen’s University Belfast, Belfast, UK; 4 Health Sciences, Brunei University London, London, Middlesex, UK; 5 Northumbria University, Newcastle Upon Tyne, Tyne and Wear, UK; 6 Newcastle University, Newcastle Upon Tyne, UK

**Keywords:** Bronchiectasis

## Abstract

Bronchiectasis is a heterogeneous lung disease. There is an increasing focus on personalised medicine in bronchiectasis, with targeted pharmacological interventions for inflammation, immunology and infection. Airway clearance techniques (ACTs) are non-pharmacological treatments used to manage bronchiectasis. Approximately half of patients with bronchiectasis perform ACTs. There have been attempts to personalise ACT prescriptions, including consideration of patient physiology, disease status and psychosocial factors. Guidelines suggest that patient preference or choice should be considered when prescribing ACTs. There is a lack of literature showing patient preference or choice being taken into consideration when prescribing ACTs in bronchiectasis. This article discusses the role of shared decision-making (SDM), the potential use of SDM for ACTs in bronchiectasis to support patient choice of and adherence to ACTs and the steps involved in designing an SDM intervention for ACTs in bronchiectasis for future research. Development and use of an SDM intervention to support patient choice of ACT in bronchiectasis may result in a patient-centred, pragmatic approach to empower patients to be actively involved in their care, improve their knowledge on the importance of ACTs and support improvement in adherence to this essential therapy.

## Introduction

 Bronchiectasis is a chronic inflammatory respiratory condition with airway dilatation, abnormal mucus production and recurrent infections.^1^

Airway clearance techniques (ACTs) are non-pharmacological treatments to facilitate mucus expectoration and are recognised as the cornerstone of management of bronchiectasis.^2^ There are a myriad of ACTs available and prescribed in clinical practice.^3^ Each ACT will have a single or combined mechanism of action that supports the mobilisation of mucus; these include but are not limited to improvement of collateral ventilation, increase of expiratory airflow velocity or a change in airway pressures and generation of airway oscillations.^4^ Factors influencing the choice of ACTs are complex and include, but are not limited to, patient preference, clinician knowledge and familiarity, access to resources/equipment and availability/reimbursement approvals particular to each healthcare system.^5 6^

To date, there are no robust clinical trials suggesting the efficacy of one specific ACT over another.^4^ The highly heterogeneous nature of bronchiectasis, the fluctuating physiological status of patients and an increasing market specifically in ACT devices,^7^ suggest it may be unlikely that one specific ACT will ever be tested and demonstrated as superior to another throughout the disease trajectory. A study by Spinou *et al*^3^ using data from the European Multicentre Bronchiectasis Audit and Research Collaboration Registry highlighted that just over half of patients with bronchiectasis perform ACTs. There was geographical variability in these data; for example, in Northern Ireland, patient-reported ACT use was 86% (n=177/205), whereas in Switzerland and Turkey, patient-reported ACT use was 11.8% and 14.9%, respectively.

Despite this, ACTs remain an essential component of bronchiectasis management, and patient adherence should be optimised. Studies have investigated barriers and facilitators to adherence in ACTs in bronchiectasis, citing lack of time, competing priorities, privacy concerns and lack of perceived consequences from non-adherence as the most common barriers to ACT adherence.^8^,^10^ Many authors and national guidelines suggest the use of shared decision-making (SDM) and patient preference when prescribing ACTs as both best practice and a potential facilitator of ACT adherence.^11^,^13^ This article aims to discuss the role of SDM, the potential use of SDM for ACTs in bronchiectasis to support patient choice of and adherence to ACTs and steps involved in designing an SDM intervention for ACTs in bronchiectasis for future research.

## Shared decision-making

SDM is an approach where clinicians and patients are encouraged to make treatment decisions together, using the best available evidence. The evidence, for example, sharing the risks and benefits for a particular treatment, should be shared in an unbiased and comprehensible format for patients. SDM encompasses an inherent association with adherence, that is, it values patient involvement, participation and autonomy in decision-making.^14^

### SDM in healthcare and healthcare education

Several national quality organisations, including the National Institute for Health and Care Excellence in the UK and the Institute for Quality and Efficiency in Healthcare in Germany, recommend including SDM as routine practice within healthcare consultations.^15 16^ These recommendations suggest the willingness of healthcare systems to transition from the previously dominant ‘biomedical model’ towards the ‘patient-centred model’ of care as a potential solution to the increasing complexity in clinical medicine.^17^

Teaching in SDM skills, quality of information and evidence base is becoming embedded in university healthcare programmes in Canada, Germany and Norway.^18^,^20^ In the UK, there is a commitment from National Health Service England to include SDM training in universities as part of undergraduate healthcare education, in addition to online and face-to-face training for existing professionals.^16^

Thompson *et al*^21^ conducted a cross-sectional survey using an online questionnaire on the use of SDM in musculoskeletal physiotherapists in the UK. Interestingly, 80% (n=39/49) stated they use SDM in routine practice; however, when asked what ‘tools’ they used for SDM, only 12% reported validated interventions such as the three-talk model or the use of a decision aid.^21 22^ Self-reported use of SDM likely over-represents actual usage, which may only be accurately identified through direct observation. This study highlights a potential gap between the education and promotion of SDM and its actual application within healthcare.

### Potential barriers to SDM in bronchiectasis

In 2008, Légaré *et al*^23^ conducted a systematic review on clinician-reported barriers to the use of SDM, citing the three most common being (1) time constraints, (2) the assumption that patients do not wish to engage in SDM and (3) the clinicians’ assumption that patients will not benefit from SDM. Over a decade later, Søndergaard *et al*^24^ conducted a study on the impact of SDM on time consumption in cancer clinics. It showed consultations were not significantly shorter in the control cohort (n=146; 22 min and 5 s) compared with the SDM cohort (n=115; 24 min and 16 s; p=0.2217). Despite this, perceived barriers to SDM remain unchanged; for example, in 2021, Hoffmann *et al*^25^ assessed 372 Australian physiotherapists’ perceived barriers to SDM implementation in practice. Their study showed time constraints (52%) and insufficiently educated or confident patients (76%) as the most common perceived barriers.^25^ It is unclear how confident and how well-trained physiotherapists are in SDM despite the perceived increase in education over time.

A systematic review by Joseph-Williams *et al*^26^ explored patient-reported barriers to the use of SDM. They found insufficient information about treatment options, and the perceived power imbalance between the patient and clinician was a significant barrier.^26^ Perceived power imbalances between patients and clinicians in medical consultations are ubiquitous in SDM literature in healthcare. For example, Sandman and Munthe^27^ debate whether some clinicians interpret SDM involving the sharing of information by both the patient and the clinician, but the final decision is still made by the clinician, following the traditional ‘paternalistic’ medical model. Lewis^28^ argued in the exact same consultation that, were the patient to make the final decision, this would suggest an ‘informed choice’ by the patient, rather than true SDM. Edwards and Elwyn^29^ suggest the focus of SDM is primarily ‘on the process of involving patients in decision-making rather than attaching importance to who actually makes the decision’.

Barriers to implementing SDM in healthcare education and practice still exist. These range from curriculum- and faculty-related, limited resources and pressure from health systems to meet quantitative metrics.^30^ There are many interventions on how to use and embed SDM in clinical practice, and it is beyond the remit of this article to explore them all; we will discuss the most commonly used SDM intervention in healthcare, decision aids.^31 32^

### SDM decision aids

Numerous SDM interventions use ‘decision aids’, which are commonly user-friendly tools, available in multiple formats.^33^ These aids present evidence-based information to patients, supporting them in making informed decisions. Decision aids are particularly suitable when multiple options are available, and the optimal decision involves considering the patient’s preferences.^34^

A recent Cochrane review concluded that across a range of chronic conditions, including diabetes, cancer and respiratory conditions, patient decision aids supported more adults to reach informed value-based choices, increased knowledge and personalised treatment compared with usual care.^33^ Increasingly, there are emerging studies investigating the use of SDM interventions on treatment adherence and clinical benefits in respiratory diseases, including chronic obstructive pulmonary disease, asthma and pulmonary hypertension,^35^,^37^ and respiratory treatments such as pulmonary rehabilitation.^38^,^40^

### Why SDM may be important for ACTs in bronchiectasis

SDM is appropriate where treatment recommendations are not definitive, and many people are likely to make different decisions based on several factors, including the information they receive (eg, the evidence base for a treatment), their lifestyle (eg, work, social or family commitments) and the availability of treatment options.^15^ These criteria represent the current prescribing practices of ACTs in bronchiectasis in the UK and Europe.^3 5^

Our systematic review by McCallion *et al*^41^ found no SDM publications in ACTs for people with bronchiectasis, highlighting a knowledge gap. Based on the systematic review of SDM interventions by Stacey *et al*,^33^ it seems highly plausible that SDM can be successfully applied to ACT in bronchiectasis. However, the need for studies on personalised treatment approaches, including SDM in bronchiectasis, has been suggested in guidelines.^1 11^ Furthermore, the multimorbidity in bronchiectasis, the wide range of ages affected and the evidence of seasonality and risk of exacerbations mean a formal study developing SDM tools will help underpin the cornerstone use of ACTs in this heterogeneous condition.^42 43^

This article has highlighted the importance of SDM in clinical practice and why SDM may be important for ACT in bronchiectasis. We will now discuss the steps and potential challenges for developing an SDM intervention to support patient choice of ACT in bronchiectasis and provide an example of how this may look and function in clinical practice.

## Steps and challenges in developing an SDM intervention for ACTs in bronchiectasis

The development of the majority of SDM interventions follows validated methodological frameworks to ensure rigour.^44^,^47^ We provide an overview of the pertinent steps required to help researchers develop an SDM intervention for ACTs in bronchiectasis, adapted from established frameworks.^44 47^ These frameworks will be used to underpin our future National Institute for Health and Care Research on the development of an SDM intervention for ACTs in bronchiectasis.

### Assessment of decisional need

The need for patients to be involved in making the decision of which ACT is most appropriate for them is highlighted in several studies and national guidelines.^1 8 41^ This need stems from the numerous ACTs available, the heterogeneity of patient symptoms and the variable lifestyle factors patients experience. The need for an SDM intervention for ACTs in bronchiectasis appears warranted, as adoption and adherence of ACTs heavily depend on patient commitment. Several studies show patients are more likely to adhere to treatment plans when they are actively involved in the decision-making process. For example, Wilson *et al*^48^ compared inhaled medication adherence and clinical outcomes in 612 adults with poorly controlled asthma. Adherence was measured using continuous medication acquisition (CMA) of patients’ inhaled medication. After 1 year, SDM resulted in significantly better inhaled corticosteroid adherence (n=204, CMA 0.67) versus usual care (n=204, CMA 0.46, p<0.0001) or clinician decision-making (n=204, CMA 0.59, p=0.029).

### Identification and engagement of stakeholders

**Figure 1:**
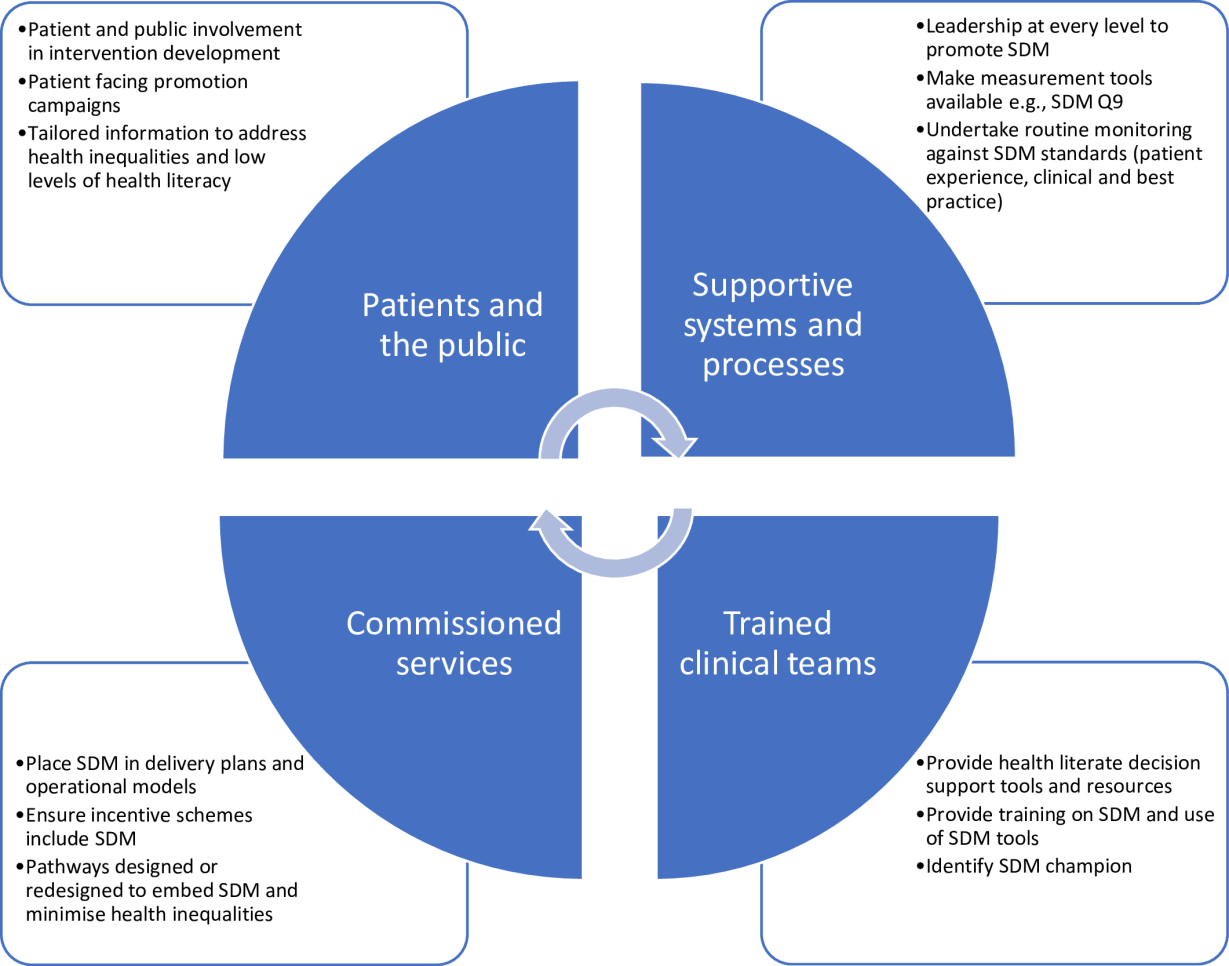
The four pillars of SDM implementation in healthcare (adapted from Joseph-Williams *et al*^68^). SDM Q9=the nine-item Shared Decision-Making Questionnaire.

There are four pillars to the successful implementation of SDM in healthcare (figure 1). The development of any SDM intervention in healthcare should include input from stakeholders within these four pillars, for example, service users, their carers, healthcare professionals and hospital or health board commissioners. The variation of stakeholders will depend on the scale and focus of the intervention(s). Studies suggest that a diverse skill set of stakeholders, for example, negotiation skills and management of expectations, can provide significant value and relevance to a project.^49 50^ The literature suggests that targeting a specific number or selecting a non-diverse group of stakeholders limits the exposure of a wider set of thoughts, potentially resulting in an intervention design not fit for purpose or unsuccessful implementation.^49 51 52^

The prescription of ACTs in bronchiectasis is largely but not exclusively conducted by respiratory physiotherapists. Key factors influencing ACT prescription in the UK and Europe are clinician experience and familiarity.^3 5^ We therefore suggest stakeholder diversity elements for physiotherapists should include a range of years working with patients with bronchiectasis in addition to age, gender and geographical location.

Efforts should be made to ensure a demographically and ethnically diverse range of views on the topic is obtained. This is important, as there are geographical and ethnic inequalities in respiratory care, including bronchiectasis, which need to be reduced.^53 54^ Additionally, there are recognised cultural barriers to SDM in healthcare, including perspectives on decision roles, personal belief systems, trust in clinicians and historical engagement in clinician-led decisions.^55^ We therefore suggest stakeholder diversity elements for patient participants could include length of diagnosis, disease severity, gender, age and ethnicity.

### Development and refinement of SDM interventions

Development of complex interventions in healthcare using co-production is more likely to be successfully adopted by both deliverers and recipients of the intervention.^49^ Co-production, in the context of healthcare, is a process where inputs used to design interventions or services are contributed by appropriate stakeholders from different organisations or areas.^56^ In this setting, this would therefore involve patients living with bronchiectasis and physiotherapists involved in bronchiectasis care.

Co-production of SDM interventions can be challenging. It relies on stakeholders sharing their concerns, participating in decisions and working often within a multidisciplinary team.^57^ These are often new roles for many participants and require careful consideration for researchers using this methodology. Common problems in co-production can include poor project design and inclusion of biased and non-representative/privileged population groups, potentially further marginalising other underserved groups.^58^ The literature provides a heterogeneous account for the number of iterative cycles used or for the management of geographical, cultural and digital literacy of stakeholders throughout intervention development.^59^,^61^ We present these common challenges and suggestions to mitigate these challenges in co-producing SDM interventions in table 1.

**Table 1:** Challenges in co-producing a SDM intervention: adapted from Bandola-Gill *et al*,^56^ Elwyn *et al*^57^ and Williams *et al*^58^

Defining roles	Sharing information	Development meetings	Financial incentives	Stages of collaboration	Dissemination and implementation
*Challenge*	*Challenge*	*Challenge*	*Challenge*	*Challenge*	*Challenge*
Researcher(s) leading development with personal views and biases. Patient and public participants viewed as ‘tokenism’.	Meeting locations and times focused on researcher and/or project deadlines. Low digital literacy participants can be excluded. Geographical spread of participants.	Limited research funding. The complexity of intervention and input required from the co-production team may be difficult to predict. Lack of clarity on most inclusive format, ideal number of stakeholders, frequency of meetings and target number usually required to attain consensus.	Limited research funding. Biased enrolment may encourage stakeholders with a lower socioeconomic status to participate in research at a higher rate than stakeholders with a higher socioeconomic status. Similar bias may relate to retired versus employed status.	Selected aspects of the project may not be appropriate for all stakeholders, for example, data analysis, academic writing for publication and dissemination in clinical environments. Earning curve may mean ‘expert patients’ full contribution cannot be realised within an initial project as they develop their skills.	Researchers may be unwilling or uncomfortable for stakeholders to disseminate project findings such as posters, and presentations or write scientific publications. Researchers may feel the intervention(s) require several levels of testing (feasibility, pilot and randomised controlled trial) which may (inappropriately) delay implementation into practice.
*Suggestions*	*Suggestions*	*Suggestions*	*Suggestions*	*Suggestions*	*Suggestions*
Clearly establish roles and responsibilities for each participant. Ensure equity of power and contribution of all stakeholders to the development process. Offer support to novice stakeholders to allow effective engagement and contribution.	Consider a hybrid approach of face-to-face meetings and online/virtual. Consider community venues outside of healthcare or academia. Use of interpreters or the availability of meeting documents in different languages.	Regular meetings ensure that stakeholders remain aligned, supporting continuous dialogue and addressing any emerging issues. Regularity or ‘intensity’ of meetings will depend on the complexity of the intervention and timelines. Research suggests between three and six meetings as a guide for 1 year project.	Appropriate funding and financial remuneration can enable more authentic and visible co-production. The National Institute of Health and Care Research provides guidance on how to appropriately fund co-production projects.	Collaboration throughout the entire research process, where possible, from conceptualisation to the implementation of the interventions. Transparent discussions with stakeholders early if any component(s) are deemed inappropriate for ‘true’ co-production.	Transparent discussions with stakeholders early on their interest or skills in planned methods of future dissemination. Early implementation of the intervention or conducting randomised controlled trials to compare against usual care should be discussed with stakeholders early and particularly after feasibility testing.

Stakeholders will have various levels of time, resources and skills available to dedicate to the process. Ensuring that these differences do not result in certain stakeholders having more influence over the final intervention design is essential.^60^ There is scant evidence detailing the ratio of different stakeholders (eg, the proportion of patients to healthcare professionals) or the level of contribution for individual stakeholders at any one stage of development.^59 62^ Morton *et al*^63^ suggest different stakeholder ratios may fluctuate between stages; for example, initial development and refinement of interventions may require greater input from service user involvement, whereas planning for implementation and evaluation may require more input from service managers and commissioners (see table 1, ‘stages of collaboration’).

### Acceptability, implementation and evaluation

It is estimated that over half of the money invested globally in research is wasted during multiple stages, including intervention design flaws and lack of dissemination or implementation.^64^ Patients’ and healthcare providers’ involvement (figure 1) and acceptability of a new healthcare intervention can have a significant influence on the implementation and sustained use of the SDM intervention.^65^ Sekhon *et al*^46^ suggest that patients and healthcare professionals can establish opinions about the potential acceptability or unacceptability of an intervention even before they undergo it. These opinions might stem from the information available about the intervention, perceived appropriateness in addressing the clinical problem, perceived suitability to the patient’s lifestyle and convenience.^66^

Exploration of these themes should be explored with patients living with bronchiectasis, their carers and healthcare professionals treating them. Evaluating anticipated acceptability and understanding possible barriers to using SDM for ACTs in clinical practice before design may pinpoint specific aspects of the intervention that could be adjusted to enhance its acceptability. Consequently, factors such as participation rates in trials and the implementation of the intervention can be addressed.^15 46^

A review exploring practical barriers to implementation of SDM in clinical practice found that insufficient resources (for both patients and clinicians) were most frequently cited.^67^ It is therefore essential that there is a focus not only on the development of an SDM intervention for ACTs in bronchiectasis but also on appropriate methods of dissemination of the intervention so that it is widely available and accessible to the population it seeks to support (table 1).

### How an SDM intervention for ACTs in bronchiectasis may work in clinical practice

In practice, an SDM intervention for ACTs in bronchiectasis could range from a simple sheet of paper illustrating the advantages and disadvantages associated with a specific number of ACTs to a more detailed patient decision aid. This more detailed decision aid could provide information on bronchiectasis and ACTs, ranging from illustrations to video formats, and include interactive questions to elicit patient values and preferences. The responses to these questions could support decision-making on which ACT to choose (eg, a preference to use an adjunct or not). These SDM interventions could be provided to patients before, during or after consultations. Potential challenges to these being adopted include developing content that is acceptable to physiotherapists and patients, appropriate language and translations and for implementation, access to appropriate resources for each consultation.

## Conclusion

SDM is an increasingly important element of clinical consultations and is an effective intervention to support treatment adherence and patient satisfaction in chronic conditions.^33^ With various ACT modalities available, an increasing market specifically in ACT devices^7^ and a heterogeneous symptom burden of mucus retention within the bronchiectasis population, it may be unlikely that one specific ACT will ever become superior to another at all stages of the patient’s disease. Development and use of an SDM intervention to support patient choice of ACT in bronchiectasis may result in a patient-centred, pragmatic approach to empower patients to be actively involved in their care, improve their knowledge on the importance of ACTs and support improvement in adherence to this essential therapy.
